# Identifying mitigation strategies of comprehensive health centers against dust hazard: a qualitative study in Iran

**DOI:** 10.1186/s12873-024-00993-0

**Published:** 2024-04-24

**Authors:** Arezoo Sarani, Asghar Tavan, Kambiz Bahaadinbeigy, Mohsen Aminzadeh, Seyed Mobin Moradi, Gholamreza Khademipour, Hojjat Farahmandnia

**Affiliations:** 1 Health Services Management Research Center, Institute for Futures Studies in Health, Kerman University of Medical Sciences, Kerman, Iran; 2 Health in Disasters and Emergencies Research Center, Institute for Futures Studies in Health, Kerman University of Medical Sciences, Kerman, Iran; 3 The Australian College of Rural and Remote Medicine, Brisbane, QLD Australia; 4 Medical Informatics Research Center, Institute for Futures Studies in Health Kerman University of Medical Sciences, Kerman, Iran

**Keywords:** Dust, Hazard, Mitigation, Health centers

## Abstract

**Background:**

Exposure to dust can disrupt healthcare services and severely affect all activity domains of the health system. The aim of this study was to explore mitigation strategies for comprehensive health centers against dust hazard.

**Method:**

The present study was conducted using a qualitative design with a conventional content analysis approach in 2023. The participants in this study were managers and staff of comprehensive health centers and experts in health in disasters and emergencies in Kerman, Bam, Regan, and Ahvaz. Data were collected through interviews. Data collection continued until data saturation. The collected data were analyzed based on the steps proposed by Graneheim and Lundman. Participants’ statements, after recording and transcribing, were categorized into semantic units. Data were analyzed by using MAXQDA software version 2020.

**Results:**

The analysis of the data with 23 participants revealed 106 Codes, 13 sub- categories and 5 main categories including: (A) reducing the impact of dust hazards, (B) management functions, (C) empowerment and performance improvement, (D) maintaining and promoting safety, and (E) Inter-sectoral coordination to implement mitigation strategies.

**Conclusion:**

The findings showed that the mitigation strategies and solutions can be used by health policymakers and planners to reduce the impact of dust hazard, empower and motivate healthcare staff, develop training protocols to enhance risk perception of the staff and members of the community, create the necessary infrastructure for adoption of effective mitigation strategies in healthcare centers to create resilience and continue service delivery.

## Introduction

Disasters refer to sudden and unexpected events resulting in loss of life and health, environmental damage, and the destruction of properties and assets. Disasters have also adverse effects on the social and economic infrastructure of a given area or country [1]. The United Nations Office for Disaster Risk Reduction (UNDRR) reported that 1.3 million people lost their lives and about 4.4 billion people were injured, homeless, displaced, or in need of emergency assistance due to geophysical and climatic disasters from 1998 to 2017; these disasters also led to economic damage amounting to $2908 billion. Most of the damages were caused by earthquakes and tsunamis. Besides, floods, storms, droughts, heat waves, and other severe weather events accounted for 91% of all these disasters [2].

The World Meteorological Organization (WMO) has defined dust as the effects of surface winds that bring a large amount of dust into the air reducing eye-level visibility (1.8 km) to less than 1 km [3]. Based on horizontal visibility, dust storms are also divided into four categories: weak dust with a visibility of less than 10 km, moderate dust with a visibility of 1 to 10 km, severe storms with a visibility of 200 to 1000 m, and very severe storms with a visibility of less than 200 m [4–6]. Sand and dust storms (SDSs) are one of the most frequently found hazards in arid areas [5–7]. SDSs contain very small particles with allergenic and polluting effects [6, 8–10]. Dust particles contain minerals, organic matter, and pathogenic microorganisms [8–10] and other elements such as aluminum, iron, potassium, magnesium, sulfur, phosphorus, sodium, manganese, barium, zinc, nickel, lead, chromium, cobalt [11, 12], and sometimes a wide variety of anthropogenic pollutants and salts depending on the source of the dust [13]. The U.S. Environmental Protection Agency has reported that dust particles less than or equal to 10 μm are respirable. Furthermore, the articles less than 2.5 μm may enter the blood causing serious consequences such as asthma, pneumonia, aspergillosis, and allergic rhinitis in the respiratory system of the affected person [14].

Air pollution caused by SDSs has caused many serious environmental and health consequences and challenges in different countries in recent years. According to WHO, cities in Iran are among the 24 most polluted cities in the world with the highest average annual PM10 concentration [15]. Air pollution causes many health, economic, and social consequences in the affected areas immediately and in the long term. Some of the immediate effects of dust storms are immediate human health problems (e.g., respiratory problems and mortality; annual and permanent product damage) and infrastructural damage to buildings and electrical, and telecommunication structures. Moreover, long-term effects of SDSs include human health problems (e.g., bronchitis, cardiovascular and other disorders), the settlement of heavy metals and salts on surfaces, and precipitation changes [16].

Following the Prevention, Preparedness, Response, and Recovery (PPRR) approach, the basic steps to disaster management to enhance the effectiveness of health response in disasters are the prevention and mitigation of effects, preparedness, response, and recovery [17, 18]. The damage prevention and mitigation stage are one of the vital stages of disaster management and involves some actions to reduce the harmful effects of disasters. Moreover, the community’s vulnerability to hazards is evaluated and some corrective measures are taken in the pre-disaster phase [19], including structural, non-structural, and functional measures [20].

The healthcare system refers to an organized group of individuals and resources that provide healthcare services to meet the needs of the target population [21, 22]. The healthcare system aims to promote, restore, and maintain people’s health through the constant provision of healthcare services [21–23]. Healthcare centers play a pivotal role in offering health services to members of the community [24, 25]. The geographical distribution of healthcare centers across the country and the easy access of people to services even in remote and less privileged regions, which are more exposed to dust hazards, are one of the main advantages of these centers. Thus, it is essential to maintain these centers to provide effective health services to the community affected by SDSs. Furthermore, in the absence of effective disaster risk management plans and strategies, the occurrence of hazards, such as dust storms, can cause many structural, non-structural, and functional challenges in these centers, disrupting all activities and services of the health system, leading to increased emergency medical dispatches due to respiratory problems, increased hospitalizations and admissions of cardiovascular and respiratory patients, and an increase in daily visits, and finally an increase in the workload of medical staff in healthcare centers [26]. Dust storms may also bring about substantial economic losses to the health system. As a case in point, these losses are estimated to be USD 66 million in Zabol, Iran, and 306 million in Iraq [27].

Since no comprehensive study has addressed mitigation strategies adopted by healthcare centers, the present study attempted to find out the mitigation strategies most frequently adopted by healthcare centers during dust hazards. The insights from this study can have some implications for policymakers and health planners to create the necessary infrastructure for the adoption of effective mitigation strategies in healthcare centers to create resilience and continue service delivery in the affected community, address the special consequences of dust hazards for the health of residents, and reduce human, financial, and social damage to the affected community.

## Materials and methods

### Study design

A qualitative study was conducted from July 2022 to March 2023 as part of more extensive research on the designing a program to mitigation of health comprehensive centers in the dust hazard. This study adopted a conventional qualitative content analysis method which is used when a researcher intends to gain a new understanding of a new field and develop a model or theory in that field [28].

### Study population, participant selection, and data collection

The participants were managers, employees, and staff working in comprehensive healthcare centers, the medical university emergency operation center, the department of environment health, department of natural resources, the disaster risk reduction office of the health department, the county health center, Iran meteorological organization, the provincial crisis committee, the technical engineering office of the university, and department of health in disaster and emergency of medical universities.

The participants (*n* = 23) were selected using purposive sampling and snowball with maximum variation in terms of gender, age, education, work experience, and occupation. Before starting the interview sessions, the researcher provided some oral instructions about the objectives of the study, voluntary participation in the study, confidentially and anonymity of the participants’ data, and their freedom to withdraw from the study at any stage. Oral informed consent was also obtained from the participants for conducting and recording the interviews. The participants were asked to specify the time and place of the interview as they wished so that they could freely and more calmly express their experiences and perceptions. Each interview was conducted individually and at a predetermined place.

One interview was conducted at the researcher’s workplace in Kerman, and other interviews were conducted at the participants’ workplaces. The interviews with the participants living in Bam and Dezful were conducted via phone calls. Each interview lasted 9 to 80 min. All the interviews were recorded, except for one interviewee who did not agree with recording the interview. Thus, the researcher transcribed the content of the interview on paper. After each interview, its content was transcribed immediately, the significant statements in the interview were underlined, and the accuracy of the data was checked and confirmed. Any ambiguity in the participants’ statements was resolved by asking for further clarification. The researcher tried not to involve her presumptions, perspectives, and prior knowledge in the interview process and not to influence the participants’ responses. The data from the interviews focused on factors effective in dust hazard mitigation in comprehensive healthcare centers to develop a dust mitigation program. During and after each interview, its content was summarized and the key statements were extracted. The participants also reviewed the texts of the interviews to confirm their accuracy.

The semi-structured interviews started with an open question. Based on the participants’ responses to the main research question, deeper and more coherent questions were asked about mitigation strategies.The open-ended questions asked from the participants included the following:

- How has your experience been in dealing with dust?
- In your opinion, what is the effect of dust hazard on the employees of comprehensive health centers?
- From your point of view, what is the effect of dust hazard on the performance plans of comprehensive health centers?
- In your opinion, what is the effect of dust hazard on the structural and non-structural vulnerability of comprehensive health centers?
- So far, what measures have been taken to mitigation of comprehensive health centers in the face of dust hazard.

Data saturation was achieved after 21 interviews. After interviewing 23 experts, the researcher came to the conclusion that saturation had been achieved and stopped sampling after conducting two more interviews to ensure that no new data was obtained.

### Inclusion and exclusion criteria

The inclusion criteria were having adequate experience with and knowledge about dust hazards and the willingness to participate in the study. The exclusion criteria were the unwillingness of the participants to cooperate and not having adequate information about the subject in question.

### Data analysis

Qualitative content analysis is a valuable method for analyzing a huge bulk of textual data in a specific context to identify the most important categories in a text. Data analysis in this study was performed by the research team who had the experience of conducting qualitative studies, at the same time as the data collection process using Graneheim and Lundman’s (2004) [29]. five-step content analysis method: determining the content and unit of analysis, determining semantic units, summarizing and abstracting, categorizing codes into subcategories, and extracting main categories from subcategories. The data analysis in this study was performed with MAXQDA-2020 software to extract the mitigation strategies.

### Trustworthiness

To check the rigor of the data, the trustworthiness criteria proposed by Guba and Lincoln [30], were used. The credibility of the data was ensured through the researcher’s prolonged engagement with the phenomenon in question. Moreover, the themes extracted from the content analysis were reviewed and confirmed by the participants, and any inconsistency was resolved through member checking, further validating the data. To check the confirmability of the findings, the transcripts of the interviews and the codification of the primary categories were reviewed by two faculty members and they confirmed the coding scheme and the extracted categories. The dependability of the data was enhanced by reporting the details of the research procedure, the decisions made, and the data collection and analysis procedures to enable the replication of the study. The transferability of the findings was confirmed by a group of people who were similar to the participants but were not the same as the participants.

## Results

### The participants’ demographic data

The participants’ demographic data to explore mitigation strategies for comprehensive health centers against dust hazard, perspective of 23 employees from inter- and intraorganizational different wards were studied, showed in Table 1.

**Table 1:** The participants’ demographic data

Participant	Job experience	Education
1	30	BSc. in environmental health
2	20	MSc. in environment, water, and wastewater
3	18	MSc. in hospital management
4	22	Ph.D. in gerontology
5	16	BSc. in environmental health
6	25	MSc. in environment
7	28	Ph.D. in health education and health promotion
8	16	BSc. in public health
9	25	BSc. in geography
10	18	MSc. in environment
11	8	BSc. in civil engineering
12	15	Ph.D. in health in disasters and emergencies
13	29	BSc. in rangeland and watershed management
14	29	MSc. in geography, planning, and development
15	20	Ph.D. in management
16	19	Ph.D. in health in disasters and emergencies
17	12	Ph.D. in health in disasters and emergencies
18	32	Ph.D. in psychology
19	20	Ph.D. in health in disasters and emergencies
20	18	BSc. in public health
21	8	Associate’s degree in family health
22	16	Ph.D. in architecture
23	7	Ph.D. in civil engineering

### Main results

Based on the results of data analysis, a total of 5 categories and 13 subcategories were obtained. After several rounds of reviewing and summarizing the data and taking into account similarities and differences, 5 main categories were obtained through the content analysis method, including (A) reducing the impact of dust hazards, (B) management functions, (C) empowerment and performance improvement, (D) maintaining and promoting safety, and (E) Inter-sectoral coordination to implement mitigation strategies, as shown in Table 2. The five major categories were in turn classified into several subcategories extracted via analyzing hand-written notes and interviews. The categories and subcategories are described in the following sections.

**Table 2:** The subcategories, categories, and themes extracted in this study

Main categories	Sub categories	Example code
Reducing the impact of dust hazards	Early warning	Determining warning levels correct forecasting dust monitoring
	Risk assessment	Identifying dust hazards preparing hazard maps
Management functions	Planning	Developing intra-organizational plans developing inter-organizational plans
	Organization	Organizational structure equipment and resources
	Decision-making	Independent diagnosis specifying measures in critical conditions
	Coordination	Intra-organizational coordination inter-organizational coordination
Empowerment and performance improvement	Promoting knowledge and awareness	Staff training public training
	Psychological support	Regular counseling sessions regular psychological screening and examinations creating motivation and reassurance
Maintaining and promoting safety	Structural measures	Retrofitting sustainable landscape engineering using hazard-compatible materials
	Non-structural measures	Using suitable doors and windows standard ventilation systems covering and protecting the components inside the building
	Protective measures	Personal protective equipment (PPE) cleaning the area cleaning the equipment
Inter-sectoral coordination to implement mitigation strategies	Reducing dust production	Developing vegetation and creating green space maintaining and protecting wetlands
	Dust stabilization	Stabilizing dust centers creating fences and windbreaks mulching

### A) Reducing the impact of dust hazards

#### Early warning

The early warning refers to the provision of effective and timely information by relevant organizations to the people exposed to hazards for hazard mitigation through the notification of warning levels, correct forecasting, dust hazard monitoring, and preparation and supply of dust hazard monitoring devices. According to one of the participants:

“*One of the challenges is the lack of a plan to specify the warning levels in comprehensive health centers. If a plan is put into action to determine the warning levels and the medical staff become familiar with this plan, the impact of dust hazards on both the staff and the health center will be decreased significantly*” (Participant 16).

#### Risk assessment

It is a process to control, reduce, or eliminate the risks identified through recognizing and understanding dust hazards and preparing a hazard map to specify the locations with hazards and vulnerabilities. One of the participants stated that:

“*The officials should not do anything to add to this problem, but they should take preventive measures, such as forming an active task force for the air pollution emergency in the governorate office. Furthermore, the officials can be alerted by the Department of Environment and universities of medical sciences*” (Participant 1).

### B) Management functions

#### Planning

Planning is a major management task to specify future activities and goals and the best way to achieve them. Planning involves the preparation of documents and the development of inter- and intra-organizational plans. One of the participants stated that:

“*Having a plan is one of the basic requirements for specifying individuals’ duties and responsibilities. We must have a predetermined plan. So that if person A is not in the organization, person B, who is the closest person to this person, can perform their duties*” (Participant 12).

#### Organization

Organizing refers to the process of dividing functions among people and groups and coordinating them to achieve a specific goal. According to one of the participants:

“*I believe that the first step for dust mitigation is to evaluate and then formulate the emergency operations plan (EOP) and the incident command system with a clear structure to specify which actions should be taken by whom, whether there is a need for any change (e.g. in managing hazardous materials), and which part of the operation should be highlighted more*” (Participant 6).

#### Decision-making

Decision-making involves choosing the best option among other options or choosing the most effective option for the organization. Decision-making includes independent diagnosis and determining actions in specific conditions. According to two of the participants:

“*We told nursing assistants to say the center is closed or not based on the same horizontal view they have because the device is not available in some counties*” (Participant 1).

“*For example, one of the decisions in the health center is to cancel visiting rural areas so we can admit a greater number of patients to be visited by the doctor*” (Participant 5).

#### Coordination

A process through which the activities of internal and Intra organizational units are aligned to achieve goals. Coordination involves intra-organizational and inter-organizational coordination. According to one of the participants:

“*The health department receives information from the air pollution measuring station and declares a warning to the crisis manager of the governorate office if the dust particles in the air exceed the standard levels and then the related officials issue public notices and make decisions about whether schools and offices should be closed and vulnerable people should avoid attending outdoor spaces*” (Participant 6).

### C) Empowerment and performance improvement

#### Promotion of knowledge and awareness

Education can play an effective role in increasing the awareness of staff and the public about potential hazards and coping strategies to mitigate dust hazards. One of the participants stated that:

“*We should teach our staff in Kerman about such severe dust storms we are facing. They should know they should not open the doors and windows during a dust storm and avoid doing things that cause the air to move from outside into the center*” (Participant 12).

#### Psychological support

Psychological support such as regular counseling sessions and psychological screening and examinations can create motivation and reassurance in the study and lead to reducing the psychological effects of dust hazards. One of the participants stated that:

“*Unfortunately, nobody pays attention to mental health. I mean, when I developed a mental problem, I became depressed. Then, I started looking for treatment. But there is no one to actively consult me and protect me from this problem*” (Participant 19).

### D) Maintaining and promoting safety

#### Structural measures

Structural measures involve building renovation and improvement, retrofitting, the use of sustainable landscape engineering in healthcare centers, and the use of hazard-compatible materials. These measures can contribute to reducing the effects of dust hazards on the structural components of the building in comprehensive health centers and the feeling of security of the staff working in the centers. According to one of the participants:

“*Dust over time may destroy the color of the structure, reduce the thickness of the structure, and weaken it. Thus, measures should be taken to strengthen the structure. For instance, epoxy and antirust materials are sprayed on the surface of the structure to make it stronger in environmental conditions*” (Participant 11).

#### Non-structural measures

Measures to mitigate the impact of dust hazards on the non-structural components of comprehensive health centers include using suitable doors and windows, standard ventilation systems, stabilization of the internal components of the building, covering and protecting the internal components of the building, and the use of sustainable landscape architecture in healthcare centers. One of the participants stated that:

“*Some of the centers where I worked have broken windows or do not have standard windows. They should replace the glass or use good materials instead of cheap materials. It is better to use standard materials such as double-glazed windows because these windows prevent the entry of dust*” (Participant 3).

#### Protective measures

Protective measures refer to taking some actions to reduce environmental impacts and damage to staff which include the use of personal protective equipment (PPE), cleaning the area, and cleaning the equipment. According to one of the participants:

“*My investigations show that surgical masks are not so much effective against dust particles but N95 masks are more effective if they are available and the healthcare staff thus wear them are less affected*” (Participant 19).

### E) Inter-sectoral coordination to implement mitigation strategies

#### Reducing dust production

The results showed that some measures that can lead to a reduction in dust production include developing vegetation and creating green spaces, preserving wetlands, and mulching, which can be performed in cooperation with relevant organizations. These measures can indirectly contribute to reducing the effects of dust hazards.

“*One of the ways to prevent the entry of dust particles into the healthcare center is to create a green space around the center using plants that are resistant to storms and wind and require less water. But there has not been a serious determination for this yet*” (Participant 1). One of the participants stated that:

#### Dust stabilization

Dust can be stabilized by stabilizing dust centers, creating fences and windbreaks, and mulching that can act as obstacles to the movement of dust particles.

“*Dust centers must be identified and managed by the provision of water rights to Urmia Lake, which is known as the center of salt dust, and the Hirmand River, which is the center of dust production, or the restoration of the Hor al-Azim wetland*” (Participant 6). One of the participants stated that:

## Discussion

The present study investigated the mitigation strategies that can be adopted by comprehensive health centers during dust hazards. The identified strategies were reducing the impact of dust hazards, management functions, empowerment and performance improvement, maintaining and promoting safety, and Inter-sectoral coordination to implement mitigation strategies.

### Reducing the impact of dust hazards

A majority of the participants suggested that one of the mitigation strategies that need to be adopted by comprehensive health centers in dust hazards is to take measures to reduce the impact of dust hazards including early warning and hazard assessment.

Merrifield et al. [31] showed that public health measures such as the development of early warning systems can improve the resilience and readiness of healthcare systems in the face of climate hazards. Moreover, the existence of these systems can have many benefits and can contribute to reducing the effects of climate hazards by influencing population behavior to reduce the incidence of adverse health outcomes including reducing exposure to air pollutants and reducing potential mortality. Early warning allows people to be aware of the dangers of dust and, subsequently, take effective precautionary measures such as the use of standard masks like N95, avoiding outdoor activities, and maintaining personal hygiene to reduce the effects of such hazards. The participants believed that hazard assessment is needed as a basic measure to reduce the effects of dust hazards in comprehensive healthcare centers. Such an assessment can provide useful information about hazards and their impact on people, property, and the environment. Allahbakhshi et al. [32] highlighted the need for constant identification and assessment of dust hazards using effective tools and continuous assessment of health centers in terms of medicines, infrastructure, and personnel to properly use all capacities for on-time response preparedness in healthcare centers. Hazard assessment plays a vital role in mitigating the effects of dust hazards and is one of the most important measures in managing this phenomenon and protecting public health. It also contributes to coming up with a better understanding of dust sources, related hazards, countermeasures, the degree of vulnerability, and effective measures to manage and control dust hazards.

### Management functions

The findings of the present study also indicated that management functions such as planning, organization, decision-making, and coordination in comprehensive health centers can be effective in mitigating the impact of dust hazards. The majority of participants in the study stated that having a well-developed intr-organizational program to mitigate the damage in comprehensive healthcare centers can contribute to enhancing the resilience of these centers during hazards. Such a program can specify the role and duties of relevant organizations and authorities in mitigating the impact of dust hazards. Middleton et al. [33] stated that special dust hazard planning to effectively use the capacities, reduce the effects of climate change, specify duties and responsibilities of relevant authorities, and develop a hazard risk management plan for vulnerable groups in different sectors, including health, food, and air transportation, can play a role in improving preparedness, resilience, risk mitigation, and minimizing the effects of dust hazards. Dust mitigation planning involves measures to put into practice dust hazard mitigation functions such as increasing public awareness, effective dust management, providing emergency and livelihood facilities, and intr-sectoral collaborations. The participants reported that the incident command system should have a standard structure to specify what actions should be taken by which people. Salehi et al. [34] emphasized that changing the working hours of employees, increasing human and organizational capacity, checking equipment by relevant experts, preventing parallel work in different departments, and specifying the duties and responsibilities of employees can effectively reduce the impact of dust hazards.

Organizing through the precise specification of roles, duties, and responsibilities of individuals and groups, optimal allocation of financial, human, and technical resources, execution of effective standards and guidelines, and accurate monitoring and evaluation can play an effective role in dust mitigation. The data from the interviews with the participants indicated that decision-making involves selecting the best alternative among other alternatives. The best alternative for organizations can involve independent diagnosis and specifying actions in specific situations.Bikomeye et al. [35] stated that strengthening evidence-based decision-making by evaluating the return periods of dust and sand storms and the engagement of all sectors, including individuals and public and private organizations in decisions and related matters and the development of the possibilities following these decisions can lead to reducing the negative effects of dust hazards on human health and increasing the adaptability and resilience of systems against such hazards. Decision-making involves dust hazard identification and analysis, impact assessment, preventive measures, interdepartmental cooperation, and effective planning to manage this phenomenon. If these decisions are made by the right people, in time, effectively, and before the occurrence of the incident, they can mitigate the impact of hazards.

Some participants in the interview stated that different departments of comprehensive healthcare centers can align their activities to reduce the risk of dust. Bikomeye et al. [35] and Fatemi et al. [36] showed that public participation, inter-sectoral coordination at private, public, regional, national, and international levels, and ensuring effective coordination and collaborations between organizations when developing programs and formulating policies are very important to improve the compatibility of systems against dust hazards. Moreover, promoting coordination between farmers, researchers, the private sector, civil society, and policymakers is effective in promoting resilience against the effects of climate change. National, local, and regional disaster management programs should be coordinated by other organizations to improve the preparedness of systems and the integration of effective responses to dust hazards. Moreover, coordination between relevant institutions and organizations can lead to more effective implementation of measures and programs to improve air quality and protect public and environmental health.

### Empowerment and performance improvement

The majority of the participants in the present study suggested empowerment and performance improvement including promotion of knowledge and awareness and psychological support can play an essential role in increasing medical staff’s awareness and risk perceptions. The majority of participants stated that training can play an effective role in increasing awareness, understanding the risk to personnel, and learning strategies to cope with dust hazards. Salehi et al. [34] showed that holding workshops, training courses, conferences, and public awareness-raising campaigns, providing education not only through television subtitles but also through social networks, news media, SMS, distribution of educational booklets and brochures in different places, educational development (general and specialized education) to empower employees, development of social networks and social awareness systems for disaster risk management, and promotion of self-management training for vulnerable people against dust hazards can lead to an increase in the risk perception and awareness of managers and employees and vulnerable populations against dust hazards and ultimately improve the level of system adaptability and resilience.

Public training can help fully understand dust hazards and show how to use different strategies and safe behaviors to reduce the effects of dust hazards. Education can help people to fully inform them to take effective measures in reducing the effects of dust hazards. Training programs should be updated continuously so that even with changes in different situations, people can effectively engage in safe behaviors. Some participants stated that psychological measures such as periodic counseling sessions, psychological screening and examinations, and creating motivation and reassurance in personnel can lead to reducing the psychological effects of dust hazards. In their study in Seoul, South Korea, Lee et al. [37] showed that counseling about health risks, alternatives for harm reduction, and prevention of harmful behaviors in vulnerable people and at-risk populations, identifying and prioritizing these groups in disaster risk management programs with a focus on the risk of suicide and mental problems during dust hazards has an upward trend. Thus, carrying out effective health interventions at the right time will lead to the reduction of the socio-economic effects and the psychological burden resulting from dust hazards and the prevention of deaths during severe dust events. Since healthcare staff are engaged in providing services during dust and sand storms and under any circumstances, they need more support and incentives to get motivated for further service delivery. Moreover, given the psychological effects of dust hazards, periodic psychological counseling and screening programs can contribute to preventing the aggravation of psychological symptoms.

### Maintaining and promoting safety

The data from the present study also revealed that maintaining and promoting safety through structural, non-structural, and protective measures can reduce the impact of dust storms.

Some of the participants suggested that the structural measures including climate-resistant reconstruction, renovation, and retrofitting of buildings and centers and the use of risk-compatible materials can contribute to reducing the effects of dust hazards on the structural components of healthcare center buildings and the sense of security of healthcare staff. Al-Ahmad et al. [38] emphasized that structures should be constructed in line with regional climate and climatic stresses. The presence of obstacles such as green belts, trees, and high-raising structures such as fences around the buildings can improve the protective, safety, and shelter effects of structures against dust hazards and provide a beautiful landscape by reducing wind speed and filtering dust particles. Moreover, constructing structures using cost-effective and quality materials with a local origin can optimize structural performance. Given that building structures are affected by severe dust storms, it is necessary to consider wind force and the use of resistant materials during building design procedures.

The participants highlighted dust mitigation strategies for non-structural components of comprehensive healthcare centers, including the use of suitable doors and windows, standard ventilation systems, stabilization of building internal components, covering and protection of the components inside buildings, and the architecture of healthcare centers in line with climatic conditions. Salehi et al. [34] recommended that closing doors and windows, using thick curtains, using double-glazed windows, staying in air-conditioned buildings, limiting windows for ventilation, and cooling the air inside the building can play a role in reducing non-structural vulnerability in the building. Since people stay a lot of time inside buildings and dust hazards have the greatest impact on the non-structural components of the building, a significant effort should be focused on developing methods to achieve an optimal indoor environment.

The majority of participants pointed to protective strategies to reduce environmental impacts and damage to personnel, including physical clothing equipment and cleaning the environment, equipment, and supplies. Allah Bakhshi et al. [32] showed that using standard and filtered masks such as N95 and if not available, using Keffiyeh on dusty days, daily cleaning, promoting urban cleanliness, and including milk, fruits, and vegetables on the diet on dusty days, can be used as an effective solution to reduce the effects of dust hazards. Protective strategies contribute to the protection of public health against the adverse effects of air pollution, and the elimination or reduction of exposure to those pollutants that are known or possibly dangerous. Thus, suitable equipment for cleaning the environment and PPE should be used in healthcare centers.

### Inter-sectoral coordination to implement mitigation strategies

Finally, the present study showed that Inter-sectoral coordination to implement mitigation strategies is effective in reducing dust production and stabilizing dust. The majority of participants stated that the measures contributing to reducing dust production and the effects of dust hazards include planting trees, providing green spaces, de-desertification, preserving wetlands, maintaining and developing vegetation, and building sand dams in collaboration with relevant organizations. Wenger et al. [39] showed that planting grasses, shrubs, or trees that are compatible and stable in water use is considered a preventive measure. Thus, more attention should be paid to the selection of species that are well-adapted to harsh conditions. As a result, reducing wind speed, trapping dust particles, and protecting against sand movement will also be effective alternatives.

The United Nations Convention to Combat Desertification (UNCCD) in the guidance entitled “Assessing and Addressing Dust Storms” [40] states that the diversity of vegetation, the installation of fences, grasses, shrubs, and the establishment of vegetation in the bed of dried lakes can play a role in controlling dust storms.

Adopting strategies such as the development of vegetation, seedling plantation, and planting native and resistant plant species can contribute to reducing dust production through the absorption and filtration of dust particles. Thus, the Natural Resources Organization must take effective measures to reduce the various effects of dust hazards. The participants also highlighted the effectiveness of mulching and creating traps and barriers as measures to stabilize dust. Similarly, other studies Ekhtesasi et al. [41], Maleki et al. [7], Middleton et al. [33] showed that stabilization of dunes and quicksand, use of fences and tree belts to stabilize quicksand, installation of panels to divert airflow, bulldozing to remove quicksand rods, and use of biological stabilizers such as tree foliage and mulching can play a role in controlling dust centers, reducing the movement of dust particles, reducing the speed of dust and sand storms, and reducing soil erosion. Stabilization of dust by reducing the movement and dispersion of dust particles can indirectly lead to the reduction of the effects of dust hazards. This requires the collaboration of the Natural Resource Organization in taking measures such as mulching and planting trees.

## Limitations

One of the limitations of this study was making arrangements with the participants to conduct the interviews, which led to an increase in the research timeframe.

## Conclusion

Given the geographical expansion of comprehensive health centers throughout the country and the easy access of people even in remote and less privileged areas, it is very important and vital to maintain these centers to provide services to the communities vulnerable to dust hazards. Hence, using effective mitigation strategies and measures against the occurrence of dust hazards can contribute to reducing the adverse effects of these hazards on the structural, non-structural, and functional components of healthcare centers. Therefore, the mitigation strategies and solutions introduced in this study can be used by health policymakers and planners to reduce the impact of dust hazards, empower and motivate healthcare staff, develop training protocols to enhance risk perception of the staff and members of the community, create the necessary infrastructure for adoption of effective mitigation strategies in healthcare centers to create resilience and continue service delivery in the affected community, address the special consequences of dust hazards for the health of residents, and reduce human, financial, and social damage to the affected community. Furthermore, continuous evaluations of vulnerability and hazards and inter-organizational cooperation and coordination with other departments including the crisis management department, environmental protection agency, the meteorology department, social and news media, ICT centers, road management centers, rescue and relief committees, regional electrical companies, traffic police, municipalities, universities/faculties of medical sciences can contribute to reducing the impact of dust hazards.

## Data Availability

The data sets generated during the current study are available from the corresponding author.
